# Assessment of Taste Attributes of Peanut Meal Enzymatic-Hydrolysis Hydrolysates Using an Electronic Tongue

**DOI:** 10.3390/s150511169

**Published:** 2015-05-13

**Authors:** Li Wang, Qunfeng Niu, Yanbo Hui, Huali Jin, Shengsheng Chen

**Affiliations:** 1 School of Electrical Engineering, Henan University of Technology, Zhengzhou 450007, China; E-Mails: niuqunfeng@163.com (Q.N.); yanbohui@hotmail.com (Y.H.); chenshengsheng518@163.com (S.C.); 2 School of Food Science & Engineering, Henan University of Technology, Zhengzhou 450007, China; E-Mail: jinhuali66@163.com

**Keywords:** peanut meal DEH, taste assessment, electronic tongue, qualitative analysis, discrimination, quantitative prediction, correlation, PCA, DFA, PLS, RBFNN

## Abstract

Peanut meal is the byproduct of high-temperature peanut oil extraction; it is mainly composed of proteins, which have complex tastes after enzymatic hydrolysis to free amino acids and small peptides. The enzymatic hydrolysis method was adopted by using two compound proteases of trypsin and flavorzyme to hydrolyze peanut meal aiming to provide a flavor base. Hence, it is necessary to assess the taste attributes and assign definite taste scores of peanut meal double enzymatic hydrolysis hydrolysates (DEH). Conventionally, sensory analysis is used to assess taste intensity in DEH. However, it has disadvantages because it is expensive and laborious. Hence, in this study, both taste attributes and taste scores of peanut meal DEH were evaluated using an electronic tongue. In this regard, the response characteristics of the electronic tongue to the DEH samples and standard five taste samples were researched to qualitatively assess the taste attributes using PCA and DFA. PLS and RBF neural network (RBFNN) quantitative prediction models were employed to compare predictive abilities and to correlate results obtained from the electronic tongue and sensory analysis, respectively. The results showed that all prediction models had good correlations between the predicted scores from electronic tongue and those obtained from sensory analysis. The PLS and RBFNN prediction models constructed using the voltage response values from the sensors exhibited higher correlation and prediction ability than that of principal components. As compared with the taste performance by PLS model, that of RBFNN models was better. This study exhibits potential advantages and a concise objective taste assessment tool using the electronic tongue in the assessment of DEH taste attributes in the food industry.

## 1. Introduction

Peanut meal, a good protein raw material, is the peanut byproduct obtained after high-temperature oil extraction; it is a plant-derived protein with a high nutritional value, the content of which can range from 40.1% to 50.9% [1,2]. Nevertheless, after extraction using high temperatures and organic solvents, this peanut meal protein is highly denatured, and its nutritional value and functionality (flavor base) decrease significantly, thereby limiting its application in the food industry [3,4]. Protein enzymolysis technology has become one of the effective methods for preparing flavor bases because it is not time-consuming; it is also harmless when directly employed to cure indigestion [5]. The peanut meal protein enzymolysis solution contains several free amino acids and small-molecule peptides, which give the enzymolysis liquid a complex taste; different free amino acid and peptide compositions render different tastes. As compared to the single enzymolysis of peanut meal, double enzymolysis can reduce the bitter taste value of hydrolysates obtained via enzymatic hydrolysis and improve the utilization rate of protein, thereby providing a new method for preparing flavor bases using protein hydrolysates.

Conventionally, sensory analysis using trained panelists has been employed to assess taste attributes in food; it is the only method that directly measures the perceived food taste intensity. However, there are some disadvantages of using subjective human sensory organs to evaluate food taste characteristics. For example, the results of sensory evaluation are influenced by subjective and objective factors and with a certain degree of ambiguity and uncertainty. Furthermore, it is expensive as panelists have to be paid for their time and effort. Moreover, it is also time-consuming to organize the training. For all these reasons, there has been increasing research into alternative objective evaluation methods, such as the use of an electronic tongue, which is based on biosensors.

Since the late 1990s, electronic tongues which use an array of multi-channel taste sensors which measure response signals characteristic of the sample solution, coupled with a signal processing unit based on pattern recognition and/or multivariate data analysis algorithms have been studied as objective taste assessment devices for the qualitative and/or quantitative characterization of compounds [6,7,8]. In principle, the electronic tongue system works in a manner similar to that of a human gustatory system. The arrays of low-selectivity taste sensors mimic the human tongue to sense the different tastes instead of using special sensors to obtain single information. To identify different tastes, the signal processing unit mimics the human nervous system to collect excited sensory signals and process the data using software. Therefore, as compared to conventional sensory evaluation, the electronic tongue has the advantages of good repeatability, high resolution, as well as rapid and facile operation. To collect comprehensive signals characteristic of the sample solution, various electronic tongue systems use different electrochemical measurement methods such as potentiometry, cyclic voltammetry, and impedance spectroscopy. After over ten years of development, the electronic tongue has already been applied widely in the food and beverage industry in applications ranging from the distinction of food varieties as well as food freshness to the prediction of food ingredients and to classification of food quality in water [9,10,11], beverages [12,13,14,15,16,17,18], wine [19,20,21], milk [22,23], or oil [24,25,26]. Moreover, electronic tongues have been shown to exhibit the potential to mimic the human tasting process and the response of a sensory panel to assess samples taste [27,28,29]. They have also been used to predict the taste intensity in pharmaceutical formulations [30,31,32,33,34,35,36,37] and hydrolysates, which are mainly bitter [38,39], and the relationship between the amount of bitter substances adsorbed onto membranes and taste sensors [40]. For example, Legin [32] has applied the electronic tongue for pharmaceutical analysis, where it could discriminate between different taste modalities of substances and its masking efficiency was found to be consistent with that obtained from a human taste panel. Rachid [33] has used an Alpha M.O.S. Astree electronic tongue to evaluate the masking efficacy of sweetening and/or flavoring agents on the bitter taste of epinephrine. First, a bitterness model was constructed with six standard pharmaceutical ingredients and then the bitterness score of different E bitartrate (EB) and EB + NMI solutions was predicted. However, thus far, relatively few studies exist on the taste assessment of protein-rich samples using electronic tongues. Newman [38,39] has used the electronic tongue to assess bitter dairy protein hydrolysates and investigated the correlation between the electronic tongue and sensory panel results (*R*^2^ of 0.98). Prediction models built using sensory, chromatographic, and electronic tongues were compared; strong correlations between these models were studied, showing an *R*^2^ from 0.78 to 0.93. Multivariate data analysis and pattern recognition methods such as principal component analysis (PCA), linear discriminate analysis (LDA), and partial least-square regression (PLS) have been increasingly applied in the abovementioned taste assessment studies. In particular, PLS regression has been widely applied for the construction of numerical prediction models from using chromatographic data to electronic tongue data in the taste assessment.

These previous studies indicate that the use of an electronic tongue may be suitable for assessing taste characteristics of peanut meal double enzymatic-hydrolysis hydrolysates (DEH). However, until now, qualitative analysis and judgment of taste characteristics in DEH have predominantly been conducted by analyzing the composition of the free amino acids and peptide species as well as by molecular weight distribution. For example, acidic amino acids or a short peptide containing acidic amino acid residues tastes umami. Glutamic acid, aspartic acid, and its amide are sour, short peptides with molecular weight less than 1000 Da are slightly salty; some short peptides are bitter, while amino acids such as Gly, Ser, Thr, Ala, and Pr are sweet. Sensory analysis is the only method to quantify the perceived different taste intensity of the hydrolysates obtained via enzymatic hydrolysis. Thus far, few studies have been conducted to demonstrate the ability of the electronic tongue to assess the taste characteristics of peanut meal DEH, which has complex different tastes.

This study aims to investigate the ability of an electronic tongue to assess the taste attributes of peanut meal DEH. The response characteristics of the electronic tongue to the six types of peanut meal DEH samples and five standard taste samples were investigated by PCA and DFA. PLS and RBF neural network (RBFNN) quantitative umami and saltiness prediction models were employed to compare the predictive abilities of the intensity of umami and saltiness and to correlate results obtained from both the electronic tongue and sensory analysis, respectively.

## 2. Materials and Methods 

### 2.1. Materials

#### 2.1.1. Peanut Meal

Peanut meal samples (produced between 2013 and 2014) were commercially purchased at random from five different manufacturers in a local market. The peanut meal sample’s nutritional ingredients are 44.70% protein, 8.28% water, 5.60% ash and 1.61% fat. The protein composition of the sample was determined by the micro-Kjeldahl method (GB 5009.5-2010, *F* = 5.46). The water and ash content was determined by the constant weight method (GB5497-85) and by combustion (GB 5009.3-2010), respectively, and the fat content was determined by Soxhlet extraction (GB5512-85).

#### 2.1.2. Chemicals

Flavor protease and trypsin used for the enzymolysis of peanut meal were purchased from Novozymes (Bagsvaerd, Denmark). Acetonitrile (chromatographically pure) used for gel exclusion chromatography (GEC) was purchased from TEDIA (Fairfield, OH, America). Monosodium glutamate (MSG), citric acid, tannic acid, sugar, and salt used as the five standard taste samples were obtained from commercial suppliers. Standard chemicals such as 1 mol/L hydrochloric acid, 0.1 MSG, and sodium chloride were supplied by Alpha M.O.S. (Toulouse, France), which had to be diluted with distilled water prior to use.

### 2.2. Methods

#### 2.2.1. Sample Preparation 

First, a 5% peanut meal solution was preheated for 10 min. Second, when the temperature of the solution reached 50 °C, 2000 μ/g of the composite enzyme (800 μ/g trypsin and 1200 μ/g flavourzyme) was added to start the enzymolysis; third, after a certain time, enzyme deactivation was performed by placing the solution mixture in a boiling water bath for 10 min. Next, after cooling to room temperature, the solution was centrifuged at 4000 r/min for 20 min; the supernatant obtained was frozen, and the solid obtained after drying was stored at −20 °C. Enzymolysis was conducted for 10 min, 1 h, 3 h, 8 h, 12 h, and 24 h. For sample analysis by the sensory panel and electronic tongue, 2% peanut meal DEH solutions of the six peanut samples were used at different enzymolysis times. The solid samples were solubilized in distilled water before testing, while samples for GEC analysis were not solubilized. Before the test, MSG, citric acid, tannic acid, sugar, and salt were solubilized in distilled water and prepared using different concentration gradients (Table 1). These standard sample solutions were selected for investigating the predominant taste observed in peanut meal DEH via analysis using the electronic tongue.

#### 2.2.2. Sensory Analysis

The sensory assess panel was organized with 30 panelists who have a professional food testing background. Their personal attributes were checked according to sensory standards (no smoking and no beverage drinking) and they were trained before providing the assessment score results. The training contents involved a questionnaire screening, taste discrimination of five standards, and a 5-point intensity scale assessment using the five basic tastes (Table 1) to aid scaling. The 5-point intensity scale is from 0 to 5, where 0 and 5 denote the least intense and most intense taste perception, respectively. Then, all peanut meal DEH samples under different enzymolysis times were provided to the panelists for sensory assessment at a room temperature at approximately 25 °C. Water was provided for cleansing the palate between tasting of different samples. All tests were performed in triplicate.

**Table 1:** Different concentration gradients of the five standard taste samples.

Standard	Concentration Gradient					
Umami (MSG)	0.1%	0.2%	0.4%	0.6%	0.8%	1.0%
Saltiness (salt)	0.001%	0.005%	0.01%	0.05%	0.1%	0.2%
Sourness (citric acid)	0.02%	0.04%	0.08%	0.12%	0.16%	0.2%
Bitterness (tannic acid)	0.025%	0.05%	0.1%	0.15%	0.2%	0.25%
Sweetness (sugar)	0.25%	0.5%	1%	1.5%	2%	2.5%

#### 2.2.3. Gel Exclusion Chromatography Analysis of DEH Samples

There is a typical relationship between the molecular weight distribution of protein DEH and its taste attributes. GEC was employed to determine the molecular weight of DEH using an Agilent protein purification system (sample volume: 20 μL, detection wavelength: 220 nm, mobile phase: 20% acetonitrile, elution rate: 0.5 mL/min); the relationship between molecular weight and retention volume is expressed as y = 6.699x − 0.393, *R*^2^ = 0.99, where y is the standard molecular weight logarithm of the peptide, and x is sample elution volume.

#### 2.2.4. Electronic Tongue 

The Astree Electronic Tongue Analyzer (Alpha M.S.O, Toulouse, France) was used for measurement. It consists of a 16-position autosampler for automatic sampling with 120 mL beakers as sample containers, a sensor array of seven different lipid membrane sensors mounted around a Ag/AgCl reference electrode, and an electronic unit for data acquisition and autosampler control. The seven sensors used to detect chemically dissolved compounds and acquire data are ZZ, JE, BB, CA, GA, HA and JB, respectively. The working principle of the electronic tongue system is as follows: when certain samples pass through the lipid membrane, they will cause a change in the membrane potential, and then the ions of samples are detected. The different sensors are composed of different lipid membranes; hence, they exhibit different sensor selectivity and potentials. The electronic unit measures all potentials between each sensor and the reference electrode and investigates the taste characteristics of the sample by analyzing the difference in potential. Before sample analysis with the electronic tongue, it is necessary to finish the start-up procedure, which consists of the three tests using each of the 80 mL standard chemical samples: conditioning, calibration, and diagnostic.

Two kinds of samples were supplied for electronic tongue analysis: 2% peanut meal DEH solutions at different enzymolysis time and five standard taste sample solutions at different concentration gradients. For electronic tongue analysis, 80 mL of the sample solutions was poured into a 120 mL beaker and placed into the electronic tongue automatic sampler. Then, the solutions were tested according to numerical order. Cleaning fluid (distilled water) and samples were placed alternately.

To ensure the accuracy and stability of the response signals by the electronic tongue sensor, the time to acquire data for each solution was 120 s, and the cleaning time was 20 s after the measurement of each solution. Data were collected every 1 s, and measurement data obtained for each solution was taken as the average of the last 5 s. To reduce the measurement error, each solution was repeatedly measured 10 times, and the last three measured values of each sensor were considered as reliable data used as input for subsequent analysis. Five duplicates of each peanut meal DEH sample were prepared from different peanut meal samples. Hence, a dataset of 90 samples for peanut meal DEH samples was supplied for analysis. Once the peanut meal DEH samples which were dried and stored at −20 °C were removed and opened, simultaneous measurement was followed by sensory analysis, GEC, and electronic tongue to ensure consistency in sample data.

#### 2.2.5. Data Processing

One-way ANOVA was conducted using SPSS 14.0 statistical analysis software with a significance difference (*p* < 0.05) for sensory assessment. PCA, DFA, and PLS were applied (SPSS Inc., Chicago, IL, USA) using the Alpha M.S.O data statistical software. RBFNN was conducted in MATLAB 7.1. Concretely, the response characteristics of the electronic tongue to DEH samples and five standard taste samples were investigated, while qualitative assessment of the taste attributes of DEH samples was performed by PCA and DFA. PLS and RBFNN quantitative prediction models were employed to predict taste intensity scores of peanut meal DEH and study the correlation between the results obtained by electronic tongue and sensory analysis, respectively.

As explained in Section 2.2.4, the total data set for the qualitative analysis of the DEH samples was 7 (sensors) × 90 (samples) matrix. Taking into account the construction of the prediction models, the reduction of the input data was important and necessary for reducing complexity, which in turn can avoid data redundancy, decrease model training time, and obtain a better prediction ability model. PCA can be used for the effective compression of data with less information loss. Two data sets were used in the PLS and RBFNN quantitative prediction models. One data set was a matrix of 7 (sensors) × 90 (samples) as before, while the other was a matrix of 4 (the first four principal component, PC, values) × 90 (samples).

To estimate the predictive ability of the PLS and RBFNN models, 3-fold cross-validation was performed, where the original 90 samples were randomly partitioned into three equal-sized subsamples. Of the three subsamples, a single subsample (30 samples) was retained as the validation data for testing the model, while the remaining two subsamples were used as training data. Cross-validation was then repeated three times with each of the three subsamples used exactly once as the validation data. The root-mean-square errors (RMSEs) from the folds were averaged. 

In the cross-validation of PLS, *R*^2^ (Test) and *R*^2^ (Prediction) need to be calculated. *R*^2^ (Test) provides a variation ratio from the explanation of the prediction variable in each response, which determines the goodness-of-fit of each model with sample data. On the other hand, *R*^2^ (Prediction) indicates how fine each predicted response was from the calculated models; it was only calculated using cross-validation. If the two *R*^2^ values are close, a fine model is built; however, if *R*^2^ (Test) is significantly lower than *R*^2^ (Prediction), prediction results are overoptimistic.

RBFNN is a three-layer forward network using the radial basis function as the activation function. It is based on the k-means clustering algorithm with two main parameters of overlap and hidden layer number, which affect network performance. After optimization of overlap and the hidden layer number with training, two types of structures in the RBF neural network were designed. One was composed of 7-8-1, where response voltages of seven sensors obtained by the electronic tongue were used as the model input neurons, eight neurons in the hidden layer, and one neuron for the prediction score of taste intensity by the electronic tongue. The other was composed of 4-6-1, where 4 denotes the first four PC values selected as the model input neurons, six neurons in the hidden layer, and one neuron as before. 

## 3. Results and Discussion 

### 3.1. Sensory Analysis

**Figure 1:**
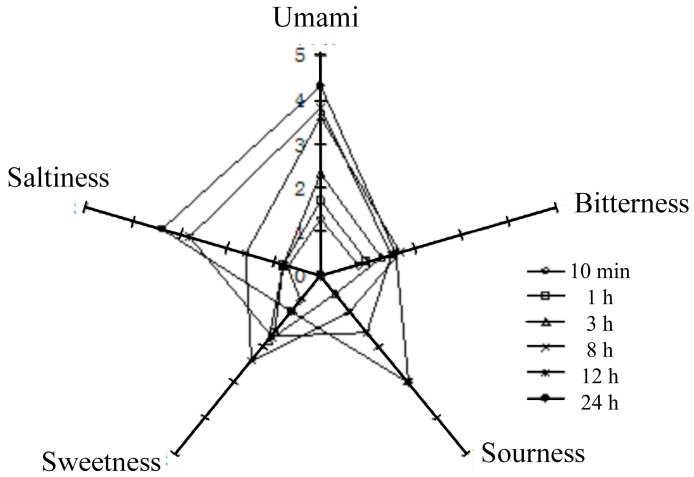
Radar distribution results of sensory analysis.

The quantitative sensory evaluation of the five taste attributes (umami, saltiness, sourness, sweetness, bitterness) of six peanut meal DEH samples (denoted as A1–A6) at different enzymolysis times (10 min, 1 h, 3 h, 8 h, 12 h, 24 h) was performed (Figure 1 and Table 2). Rather than a single taste, five complex tastes including umami, saltiness, sourness, sweetness, and bitterness were observed for the DEH samples. Among these tastes, the intensity of umami was the maximum (score 1.3–4.2), followed by that of saltiness (score 0.6–3.4); the intensities for both sourness and sweetness were weak, while the intensity of bitterness was the weakest (score 0.7–1.8).

As shown in Table 2, the trends of umami and saltiness gradually increased within 24 h with the progression of enzymolysis time. Sensory analysis showed that the umami score was the highest for samples A1–A6, with umami being predominant.

**Table 2:** Characteristics and taste intensity scores of peanut meal DEH, which were used to compare the taste attributes obtained by the electronic tongue.

Sample	Enzymolysis Time (min)	Degree of Hydrolysis (DH) (%)	Protein Extraction Rate (%)	Ratio of Different Relative Molecular Weight Peptides and the Total Peptides % (Da)			Taste Intensity Score	
				<1 k	1–3 k	>20 k	Umami	Saltiness
A1	10	4.8	61	6.38	11.64	37.72	1.3	0.6
A2	60	8.2	63	13.27	14.22	25.98	1.7	0.7
A3	180	11.0	78	14.6	16.8	21.96	2.2	0.9
A4	480	15.2	72	20.79	16.86	20.73	3.6	1.6
A5	720	17.1	71	24.02	16.77	15.4	3.8	2.8
A6	1440	20.4	68	26.34	20.36	10.23	4.2	3.4

### 3.2. Analysis of Taste Attributes by Gel Exclusion Chromatography

GEC was conducted to obtain the molecular weight distribution of peanut meal DEH (Table 2). The peptides with molecular weight greater than 20 kDa rapidly reduced as enzymolysis time progressed, while smaller-molecular-weight peptides (<1 kDa) gradually increased. For instance, at an enzymolysis time of 1440 min (24 h), the content of the peptides with molecular weight greater than 20 KDa decreased from 46.88% to 10.23%, while the content of peptides with molecular weight less than 1 KDa gradually increased from the original 0.99% to 26.34%, because trypsin (endonuclease) rapidly hydrolyzed the protein to macromolecular peptide, and the compound flavor enzyme (including endonuclease and exonuclease) continuously hydrolyzed the macromolecular peptide to small-molecule peptides or free amino acids during enzymolysis. The small peptides with molecular weight less than 1 kDa clearly exhibited umami and saltiness (small peptides with molecular weight 1 k–3 kDa have umami-enhancing effect). Hence, from the analysis of the taste attributes by GEC, peanut meal DEH predominantly exhibits umami, and with increasing enzymolysis time, the umami taste increases. These results are consistent with those reported by *Yamada and Nishimura* as well as with former sensory analysis.

### 3.3. Representation of Sensor Response of the Electronic Tongue

**Figure 2:**
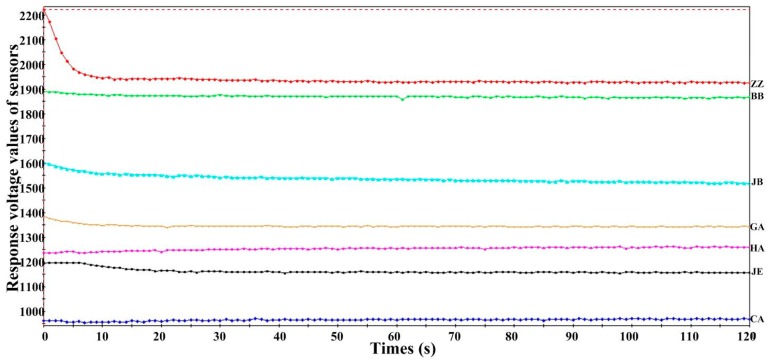
Response curves of the seven sensors to Sample A6.

Further objective analysis of the DEH samples was performed using the electronic tongue. Figure 2 shows the response curves of ZZ, JE, BB, CA, GA, HA, and JB to sample A6 (enzymolysis time 24 h). The X- and Y-axes denote the data acquisition time (120 s) and response voltage values of the seven sensors, respectively. The sensors exhibited good stability and repeatability with respect to the measured signal, except for the variation of the acquired data before 30 s.

**Figure 3:**
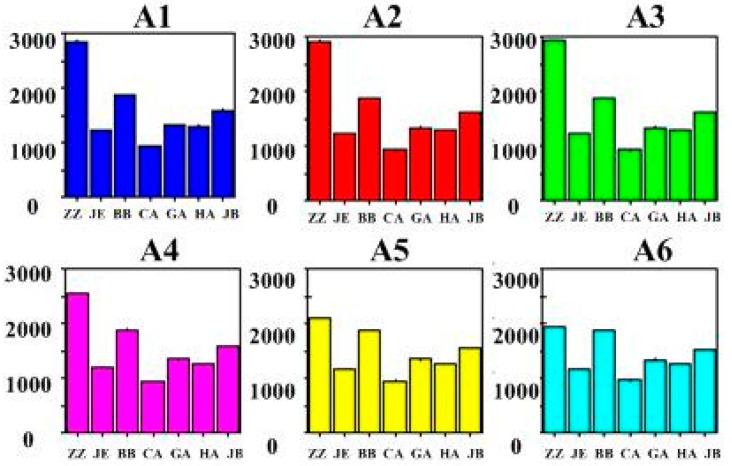
Bar graphs of the response values of sensors to all samples.

Figure 3 shows bar graphs of response intensity of the seven lipid membrane sensors to samples A1–A6. The X- and Y-axes denote each sensor and the final stable voltage response values of the sensors, respectively. The different colors represent different samples. Each sample was measured five times. Sensors ZZ, BB, and JB exhibited a higher response intensity, while CA exhibited the lowest response intensity. Moreover, JE, GA, and HA exhibited a similar response intensity.

The relative standard deviation (RSD) values of the seven sensors in the DEH samples and the five standard taste samples were less than 6%, which indicates that the difference in experimental data obtained by the electronic tongue was small and exhibits good reproducibility.

### 3.4. Analysis of Five Taste Attributes of DEH Samples 

**Figure 4:**
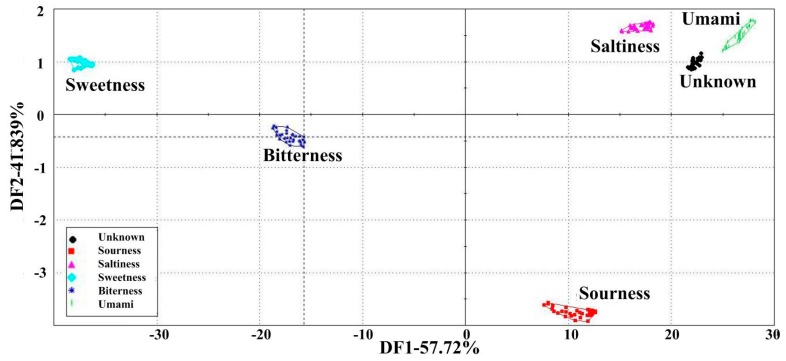
Analysis of the taste attributes of the five taste samples and peanut meal DEH samples (unknown samples was peanut meal DEH at enzymolysis times of 10 min, 1 h, 3 h, 8 h, 12 h, 24 h. Umami: 0.1%–1.0% MSG; saltiness: 0.001%–0.2% salt; sourness: 0.02%–0.2% citric acid; bitterness: 0.025%–0.25% tannic acid; sweetness: 0.25%–2.5% sugar). Data were processed by DFA.

DFA can be a useful tool for differentiating the individual to which each colony is affiliated; DFA was mapped using the response voltage values of the seven sensors for each sample as data for calculating a set of new variables called discriminant factors. Each sensor response voltage value was decided according to the average of the last three measurements of the sensor of each sample. From Figure 4, DFA showed a total contribution of 99.56% with the contribution from the first two discriminant factors being 57.72% and 41.839%, respectively, with a Discrimination Index (DI) value of 98. All samples were separated into six distinct clusters. Five standard tastes (umami, saltiness, sourness, bitterness, and sweetness) were successfully discriminated. The space within each sample was close, while the distance between the two groups was large, indicating good data repeatability and a clear distinction between different samples. All six peanut meal DEH samples as unknown samples were projected into five tastes in the DFA map. From the cluster formations in the DFA map, the position of the peanut meal DEH samples cluster (unknown cluster) was relatively close to the umami and saltiness clusters, with the position being closer to the umami cluster. The unknown cluster was at a farther distance to the sourness cluster, and the distance between the sweetness and bitterness clusters was the farthest.

Each point in each cluster represents each replicate sample measurement by the sensor array, indicating that peanut meal DEH samples exhibited characteristics similar to umami and saltiness. This result is in agreement with those obtained from sensory analysis and GEC. Samples A1–A6 mainly exhibited umami and saltiness, followed by sourness, while the intensities of both sweetness and bitterness were the weakest. Umami was the most prominent taste of peanut meal DEH samples. The DFA map from the electronic tongue showed that it can assist and even replace human sensory analysis.

**Figure 5:**
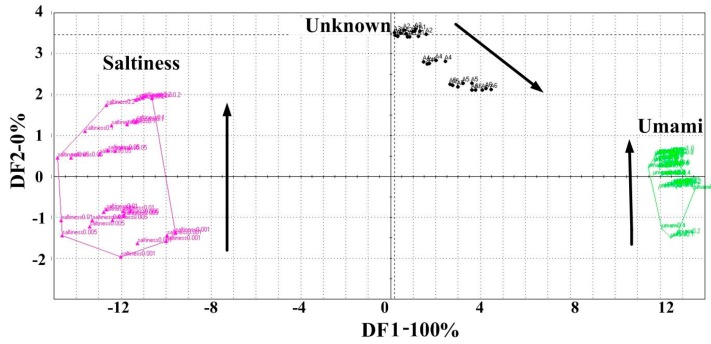
Evaluation of taste attributes of the three samples (Unknown peanut meal DEH samples under 10 min, 1 h, 3 h, 8 h, 12 h, 24 h; Umami: 0.1%–1.0% MSG; saltiness: 0.001%–0.2% salt).

In addition, the lack of discrimination among samples of different concentration gradients in the umami and saltiness clusters as well as from samples of peanut meal DEH indicated that further research is required for the discrimination of these three taste samples. The discrimination of the three taste samples with different taste attributes was also mapped with DFA using the same data extraction method as shown in Figure 4. DFA (Figure 5) showed a complete 100% contribution with the first discriminant factor (DF1) with a DI value of 100. It indicated that the DFA map can represent the response data of all samples using the electronic tongue. All samples were separated into three distinct clusters. Three taste samples—umami, saltiness and peanut meal DEH samples—were successfully discriminated. The saltiness cluster was located at the left edge of the map, the umami cluster was located at the right edge of the map, and the peanut meal DEH (unknown cluster) was located between them, albeit closer to the umami cluster. Thus, the relationship between their relative positions on this map demonstrated a strong correlation with their taste attributes perceived by human. From the saltiness cluster, the six samples exhibiting saltiness (0.001%, 0.005%, 0.01%, 0.05%, 0.1%, and 0.2%) were separated from each other, indicating that the electronic tongue can discriminate different concentrations with the same taste. DF2 in the map approximately coincided with the direction of intensity change of saltiness in samples. The arrow on the map represents the increasing direction of the intensity of saltiness. Similar results were observed for the umami cluster. Six umami samples (0.1%, 0.2%, 0.4%, 0.6%, 0.8% and 1.0%) were separated, and the direction of the arrow on the map represents the increasing intensity of umami. For peanut meal DEH samples as projected into the map as unknown samples exhibited mainly two taste attributes, it located in a direction shown with an arrow direction representing the increasing enzymolysis time. The arrow direction is also called taste intensity increase direction because according to the previous analysis, the taste intensity increased with enzymolysis time. The first three samples of peanut meal DEH were not distinguished probably because of the low concentration of the prepared DEH solution. The lack of discrimination among these three samples indicated the necessity to search for more supervised methods for the quantitative analysis of peanut meal DEH samples, such as PLS or ANN.

The results from the above analysis demonstrated that the electric tongue has the potential to qualitatively assess different taste samples. The location of the different samples indicates the taste similarity degree. In the same cluster, the location can reflect different taste intensities and can regularly change with changing concentration.

### 3.5. Discrimination of Different DEH Samples by PCA and DFA

**Figure 6:**
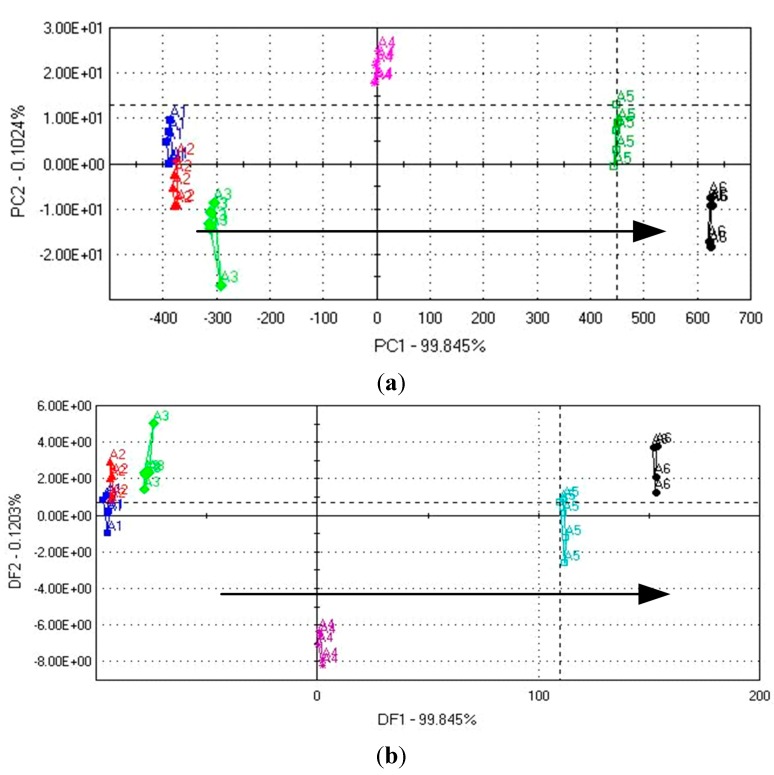
Taste discrimination of different peanut meal DEH samples (A1–A6) (**a**) score map obtained by PCA; (**b**) score map obtained by DFA.

Because of the satisfactory taste trend observed in the previous analysis, the whole set of peanut meal DEH samples was further analyzed using only the electronic tongue, and data were processed by PCA and DFA to obtain consistent taste assessment results using different data processing methods (Figure 6). Figure 6a shows the distribution of the peanut meal DEH samples along the first two new coordinates (PC1 and PC2): an accumulated contribution of 99.94% was observed with a DI value of 92. The high contribution value indicated that nearly all the data from the original sensors are presented only by the two new functions. As can be seen, except samples A1 and A2, all other samples were successfully discriminated by analysis with PCA coordinates. The arrow direction on the map represents the increase in taste intensity. Figure 6b shows similar results by DFA analysis. It shows the distribution of the peanut meal DEH samples along the first two new coordinates (DF1 and DF2): an accumulated contribution of 99.96% was observed with a DI value of 94. As can be seen, except samples A1 and A2, all other samples were discriminated by analysis of DFA coordinates. The arrow direction on the map also represents the increase in taste intensity. DFA is significantly better than PCA because it can leave some samples as unknown, as compared to the expected ones, for projection into the built model to predict classes rather than use all samples to perform classification. Hence, the actual performance of the DFA classification model can be assessed.

The analysis result showed that the electronic tongue demonstrates potential for the qualitative assessment of the taste intensity of the different peanut meal DEH samples. The samples regularly varied according to the PC1/DF1 direction, which was the direction of increasing enzymolysis time. The two methods of the electronic tongue showed consistent taste assessment results. It was also in agreement with the results obtained from sensory analysis and GEC. The intensities of both umami and saltiness of the peanut meal DEH samples (A1–A6) increased with enzymolysis time, thereby resulting in the increase of the total taste intensity.

### 3.6. Quantitative Taste Prediction of DEH

The taste attributes of peanut meal DEH were complex because a large amount of free amino acids and small-molecule peptides was present in the protein enzymolysis solution. Because of the different composition of free amino acids and peptides, the enzymolysis solution exhibits different tastes. From the information stated above, substances that were soluble in water exhibit umami and saltiness, which was easily detected by sensors; they were the main two taste attributes; also, their intensity scores for peanut meal DEH were predicted by the electronic tongue and were found to be comparable to those obtained by sensory analysis.

To assess whether the electronic tongue was suitable for the prediction of umami and saltiness in peanut meal DEH, it is essential to correlate between prediction scores obtained by the electronic tongue and actual scores obtained by sensory analysis. PLS and RBFNN prediction models were employed. Unlike previous studies that focus on the extraction of qualitative information, herein two quantitative models were built. In each model, two input datasets were extracted for model construction. One type of input variable was selected with the response voltage values obtained from the seven sensors of the electronic tongue, and the other type was the first four PC values. That is, the scores assigned for peanut meal DEH by sensory analysis were modeled from the data of sensors voltage response or from the data of the first four PC values.

#### 3.6.1. PLS Taste Prediction Model

**Figure 7:**
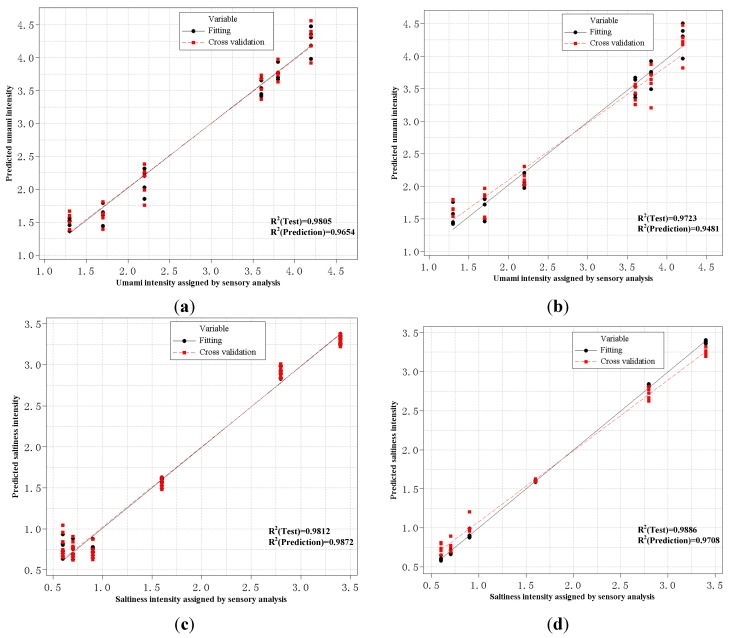
PLS model prediction of intensity scores by the electronic tongue against actual scores as scored by sensory analysis. (**a**) umami intensity scores using sensors voltage response values as model input; (**b**) umami scores using the first four PC values as the model input; (**c**) saltiness scores using sensors voltage response values as the model input; (**d**) saltiness scores using the first 4 PC values as the model input.

Figure 7a,b show the correlation between the predicted scores by PLS using the electronic tongue and those obtained by sensory analysis with respect to the intensity of umami in peanut meal DEH samples. PLS regression was built by using the response voltage obtained by the seven sensors of the electronic tongue for the peanut meal DEH samples and the scores obtained by sensory analysis. As stated before, the average of last three measurements of each sample was used as the response voltage value of each sensor. Table 3 lists the prediction formula, and Figure 7a shows the PLS regression results. A good correlation was observed between the intensity scores of umami predicted by the electronic tongue and the actual scores obtained by sensory analysis with a high *R*^2^ (Test) value of 0.9805 and *R*^2^ (Prediction) value of 0.9654 in the cross-validation test. The two *R*^2^ values are similar, indicating the effectiveness of the taste prediction model: a satisfactory trend was obtained with the regression line being close to the theoretical line. The RMSE of prediction was 0.72374. Then, PLS regression was built by using the first four PC values as independent variables and the scores obtained from sensory analysis. A better trend was observed between the predicted intensity scores of umami and the actual scores obtained by sensory analysis with a high *R*^2^ (Test) value of 0.9723 and *R*^2^ (Prediction) value of 0.9481, albeit a relatively high RMSE of 1.024. From the above analysis, the PLS prediction model using the response voltage from the seven sensors of the electronic tongue as model data input was better than by using the first four PC values. This indicated that the model constructed by using the first four PCs is disadvantageous in that compressed data information obtained by sensor detection.

The same experimental procedure was performed to predict the intensity of saltiness in peanut meal DEH by using the PLS model. Table 3 lists the prediction formula for saltiness, and Figure 7c,d show the resultant PLS using input data obtained from the response voltage from the seven sensors of the electronic tongue or the first four PC values. A strong correlation was observed between the predicted scores by the electronic tongue, obtained from the voltage response data, and the actual scores for saltiness obtained by sensory analysis with *R*^2^ (prediction) and *R*^2^ (Test) of 0.9812, 0.9872 and a relatively low RMSE of 0.32548 (Figure 7c). The resultant PLS regression using the first four PCs as model data input exhibited a relatively worse correlation than the former using the voltage response data with two *R*^2^ of 0.9886, 0.9708 and an RMSE of 0.45234 (Figure 7d).

**Table 3:** PLS models for prediction of taste intensity scores of DEH.

Taste	Input Data	Prediction Formula
umami	voltage response	Y = 76.2667 + 0.0029x1 − 0.0412x2 + 0.0303x3 − 0.0010x4 − 0.0207x5 − 0.0121x6 − 0.0280x7
	first 4 PC values	Y = 2.8 + 0.00256PC1 + 0.01402PC2 − 0.03365PC3 − 0.03814PC4
saltiness	voltage response	Y = −29.9924 − 0.0065x1 + 0.0302x2 − 0.0051x3 + 0.0009x4 − 0.016x5 + 0.0245x6 + 0.0068x7
	first 4 PC values	Y = 1.5 + 0.00266PC1 − 0.0246PC2 + 0.02367PC3 + 0022PC4

x1–x7 represented response voltage values from sensor1–7, respectively (x1: ZZ; x2: JE; x3: BB; x4: CA; x5: GA; x6: HA; x7: JB).

From the above analysis of taste prediction models, all four PLS models for taste intensity scores prediction of peanut meal DEH exhibited a good correlation with actual scores obtained by sensory analysis. The two PLS models used to predict the intensity scores for saltiness had stronger predictive capabilities with lower RMSE than those for the intensity scores for umami. In the same prediction of taste intensity scores, the PLS model for the prediction of taste intensity constructed with data from the response voltage values of the seven sensors was better than that constructed using data from the first four PCs with both higher correlation coefficients and relative lower RMSE as no detected data was lost.

#### 3.6.2. RBFNN Taste Prediction Model

RBPNN was applied to further predict the taste intensity scores by the electronic tongue as compared to linear methods caused by its high performance. The two types of input data for RBFNN were selected to be the same as those for PLS. First, the k-means clustering algorithm was adopted to determine a proper data center for the radial basis function of the hidden layer nodes and then an appropriate overlap value and the number of hidden layer nodes were selected using different combination of the two parameters. The optimization range of overlap was 1–3, while the number of hidden layer nodes was 4–10. The optimization goal was the minimum RMSE value of RBFNN. The prediction error results showed that the error of the models decreases with increasing number of hidden layer nodes. However, after the hidden layer nodes increased to a certain number, “overfitting” was observed, leading to an increase in error. Hence, the optimum number of hidden layer nodes for the two types of constructed RBFNN models was determined according to the average minimum RMSE value after three times validation. In one construction of 7-8-1, the lowest average RMSEs of 0.139 and 0.1209 were predicted for umami and saltiness, respectively, with an optimized overlap of 2; the optimum number of the hidden layer nodes was 8. On the other hand, for the other construction of 4-6-1, the lowest average RMSEs of 0.1533 and 0.1793 were predicted for umami and saltiness, respectively, with an overlap of 2, and the optimum number of hidden layer nodes was 6.

Table 4 summarizes the prediction results. It lists the RMSE, maximum error, mean absolute error (MAE), and *R*^2^, and rows having different taste attributes and different input data (using sensor voltage responses and four PCs). It also shows PLS results for comparison.

The prediction ability of the RBFNN model can be seen directly in Table 4. It shows a comparison of the prediction error for umami or saltiness assigned by using the response voltage values from the seven sensors and the first 4 PCs values; an *R*^2^ of 0.994 and RMSE, Maximum error and MAE of 0.139, 0.1531 and 0.13, respectively, was better than that by the first 4 PCs values, for which an *R*^2^ of 0.992 and RMSE, Maximum error and MAE of 0.1533, 0.2738 and 0.14, respectively. Similarly, the model prediction results for the intensity of saltiness were shown. A strong correlation was observed between the scores predicted by the electronic tongue using the response voltage data and the actual saltiness scores obtained from sensory analysis with an *R*^2^ of 0.998 and RMSE, Maximum error and MAE of 0.1209, 0.2162 and 0.115, respectively. The latter also had an excellent correlation by using the first 4 PCs as model data input with an *R*^2^ of 0.996 but with a relative higher error of 0.1793, 0.2275 and 0.169, respectively.

**Table 4:** Comparison of the model for the ability to predict different errors and *R*^2^ for DEH.

Models	Taste Prediction	Input Data	RMSE	Maximum Error	MAE	*R*^2^
PLS	umami	voltage response	0.7237	0.5056	0.222	0.9805/0.96544
		the first 4 PCs	1.024	0.7044	0.246	0.9723/0.9481
	saltiness	voltage response	0.3255	0.241	0.117	0.9812/0.9872
		the first 4 PCs	0.4523	0.4965	0.119	0.9886/0.9708
RBFNN	umami	voltage response	0.139	0.1531	0.13	0.994
		the first 4 PCs	0.1533	0.2738	0.14	0.992
	saltiness	voltage response	0.1209	0.2162	0.115	0.998
		the first 4 PCs	0.1793	0.2275	0.169	0.996

The assessment results using RBPNN model to predict taste showed that the results obtained from all four RBFNN prediction models well correlated with those obtained from the actual sensory analysis scores. In the same prediction of taste intensity, RBFNN with model input data from the response voltage values of seven sensors exhibited better ability to predict scores than that with model data from the first 4 PCs, caused by the relatively lower RMSE. In the study, RBFNN exhibited the best ability to predict the intensity for saltiness using the input data from the response voltage value of seven sensors, with the lowest RMSE of 0.1209.

A comparison of the taste assessment by the RBPNN and PLS prediction models shown in Table 4 indicated that RBPNN exhibited a stronger correlation with higher correlation coefficients and better taste prediction ability with a relatively lower error than those of PLS. For the construction of the two taste prediction models, using the response voltage values of the sensors of the electronic tongue, as compared to the first four PCs, was better.

However, despite the good prediction results obtained with both PLS and RBFNN, it is necessary to highlight that prediction results may be overoptimistic if some of the measurements in similar hydrolyzate solutions prepared from similar peanut meal samples were used both for calibration and validation. 

## 4. Conclusions

Qualitative and quantitative assessment of taste attributes of peanut meal DEH samples using an electronic tongue was attempted. Concretely, the electronic tongue was used for the qualitative assessment of the taste attributes of peanut meal DEH using PCA and DFA, and the quantitative taste was evaluated by the scores predicted by employing PLS and RBFNN models. The two taste scores from the prediction models were compared on aspects of predictive abilities of umami and saltiness intensity and the correlation between electronic tongue and sensory analysis. The results showed the electronic tongue could distinguish five tastes with different concentrations and different peanut meal DEH, which is an easy and visual indicator of the taste attributes shown in the map. Both prediction models used for quantitative assessment can evaluate taste scores in peanut meal DEH. As compared to the PLS prediction model, the RBPNN prediction model showed a stronger correlation with higher correlation coefficients and better ability to predict taste with a relatively lower RMSE. Moreover, the results further proved good consistency with those obtained by sensory analysis and gel exclusion chromatography, indicating that the electronic tongue is an effective tool for the assessment of complex taste attributes as it involves a less sample preparation time and short analysis time. Furthermore, this study demonstrated applications of the electronic tongue in the assessment of hydrolysates with high bitterness or unpleasant tastes such as single enzymatic-hydrolysis hydrolysates. The study provides a new taste assessment tool for the preparation of flavor base with peanut meal DEH in the food industry. Finally, future efforts may involve further construction of more models, validation and correlation between the electronic tongue, and sensory analysis or other chemical methods with increasing various peanut meal DEH samples under different types of enzymolysis, as well as taste assessment to mimic the human tongue as better as possible.
